# The Effect of Aerobic Exercise on Speed and Accuracy Task Components in Motor Learning

**DOI:** 10.3390/sports7030054

**Published:** 2019-02-26

**Authors:** Håvard Stranda, Monika Haga, Hermundur Sigmundsson, Håvard Lorås

**Affiliations:** 1 Department of Neuromedicine and Movement Science, Faculty of Medicine and Health Sciences, NTNU—Norwegian University of Science and Technology, 7491 Trondheim, Norway; havard.stranda@gmail.com (H.S.); monika.haga@ntnu.no (M.H.); 2 Department of Psychology, Faculty of Social and Educational Sciences, NTNU—Norwegian University of Science and Technology, 7491 Trondheim, Norway; hermundur.sigmundsson@ntnu.no; 3 Department of Sport Science and Physical Education, Reykjavík University, IS-101 Reykjavik, Iceland; 4 Department of Sport Science & Physical Education, Faculty of Teaching, Art and Culture, Nord University, 7600 Levanger, Norway

**Keywords:** acute exercise, motor learning, retention, speed, accuracy

## Abstract

Acute exercise has an influence on human cognition, and both theoretical approaches and previous investigations suggest that the learning process can be facilitated. A distinction has been made however, between the predominately positive effects on task speed compared to both the negative and null effects on aspects of task accuracy. The aim of this study was to investigate the effect of moderate-intensity aerobic exercise conducted before each practice trial (3 × week) for a period of four weeks, on speed and accuracy components in a novel keyboard typing task. To this end, young adults (*n* = 26) where randomized to a non-exercise resting group (control) or an exercise group (ergometer cycling at 65% of age-predicted maximal heart rate). Immediately after exercise or resting, participants practiced keyboard typing through specialized online software for a total of 2 h across the study period. All participants improved their speed and accuracy in the keyboard typing task. At 7-day retention, no differences were found between groups. Thus, the degree of improvement on both speed and accuracy task components was not significantly different between the exercise and control group. Further studies are warranted to establish the specific relationship between aerobic exercise and task components in motor learning and retention.

## 1. Introduction

It has been well established through a comprehensive line of studies across many decades, that acute exercise can exert an immediate positive effect upon human cognition, for example the task a participant engages in after physical exercise is considered to be facilitated and improved [1]. Indeed, several systematic reviews with meta-analysis clearly indicate that there is ample evidence for small beneficial effects (effect sizes at *d* ≤ 0.20) on cognitive task performance shortly following the termination of acute aerobic exercise [2,3,4,5]. These research efforts investigating the effects of acute exercise upon cognition are motivated by the possibility for developing exercise interventions targeted at schoolchildren [6,7], older populations [8] and various patient groups [9,10].

The evidence thus suggests that activation of the entire body, which produces systemic changes in physiological functions, can facilitate processing in various cognitive tasks [11]. Acute exercise is also hypothesized to improve memory and learning tasks in a time-dependent fashion by priming the molecular processes involved in the encoding and consolidation of newly acquired information [12]. Indeed, a meta-analysis targeted at studies with memory tasks as the dependent measures of cognitive performance by Roig, Nordbrandt, Geertsen & Nielsen (2013) reported that regardless of the type of memory assessed, pooled data indicated that acute cardiovascular exercise generated a moderate effect upon memory (standardized mean difference (SMD) = 0.22). After splitting the studies into measures of short-term or long-term memory. However, the meta-analysis indicated a large effect (SMD = 0.52) of acute cardiovascular interventions on long-term memory and a non-significant effect (SMD = 0.07) on short-term memory. The authors thus concluded that acute exercise seemed to produce moderate to large effects on long-term memory and more moderate effects on short-term memory [13].

In addition to the abovementioned diversity of results in previous studies on cardiovascular exercise and memory, a meta-analysis conducted by McMorris, Sproule, Turner, & Hale (2011) on the effect of acute intermediate intensity exercise on working memory (WM) tasks indicated dissimilar effects on components of speed and accuracy [5]. The former, which refers to the response/reaction time and/or processing speed, appeared to be substantially improved (*g* = 1.41) after an exercise bout conducted at intermediate intensity (50–75% of maximal volume of oxygen uptake, VO_2MAX_). The component of accuracy (or task performance/errors) in a working memory task on the other hand, was negatively (*g* = −0.40) affected by similar exercise bouts. A later meta-analysis by McMorris & Hale (2012) demonstrated a similar accuracy-speed distinction in studies that have applied other cognitive tasks. Cardiovascular exercise conducted at low to high intensity demonstrated no significant effect on accuracy, with a pooled non-significant mean effect size at *g* ≤ 0.14. In measurements of speed, low and high intensity exercise showed non-significant effects while moderate intensity exercise demonstrated a significant mean effect size (*g* = 0.50). Importantly, these results cannot be attributed to a speed-accuracy trade-off, as results for cognitive tasks which did not measure both speed and accuracy showed similar results for accuracy as did tasks in which a speed-accuracy tradeoff was possible. Furthermore, several studies in the meta-analysis demonstrated a significantly faster speed of processing with no detrimental effect on accuracy [4]. In studies with measures that jointly capture both speed and accuracy, results indicate the facilitation of cognitive function in response to both moderate and high-intensity aerobic exercise [14,15].

There appears to be a paucity of studies that have jointly investigated this speed-accuracy distinction by examining the effect of acute cardiovascular exercise on specific learning tasks. The current base of studies has predominantly applied perceptual-motor tasks that require tracking patterns presented on a screen. Across these studies, evidence suggest that acute exercise conducted at high-intensity [16,17,18] can facilitate the learning and retention of accuracy in motor tasks (see [19] however, for contrasting results). Snow et al. (2016) and Singh et al. (2016) however did not find improved learning and retention of motor accuracy after moderate-intensity exercise [20,21]. As for the speed-related task components, the current base of studies suggests that moderate-intensity aerobic exercise has generated positive [22] as well as no effect on motor learning/retention [20]. With respect to high intensity exercise, studies have demonstrated both positive [23] and negative effects [24] on motor speed.

It thus appears to be a distinction in the current literature with regard to the effects of acute exercise on speed/accuracy in perceptual-motor learning and cognitive measures such as working memory, in which data on the former suggest that retention of accuracy-related components can be facilitated. In the latter case however, information-processing speed is predominantly improved. The contention that acute aerobic intermediate-intensity exercise has a strong effect on the speed of response is as would be expected from theoretical perspectives on exercise-cognition. In what has been termed the cognitive-energetic approach, it is postulated that an individual’s level of arousal (i.e., the physiological and psychological state of being awoken/stimulated) is a key component in the relationship between exercise and cognition [25,26]. Acute exercise increases physiological arousal that in turn increase the availability of cognitive resources. This is further hypothesized to particularly facilitate tasks that have a high degree of complexity and require substantial mental effort [27,28]. Thus, the level of arousal corresponds to the amount of available cognitive resources such as attention and focus that can be engaged during a learning task. A general prediction is that under-aroused and over-aroused individuals demonstrate lower performance levels, whereas the most pronounced performance effects are expected in a moderately aroused state [25,29]. The cognitive-energetic approach however does not seem to provide any explanation for a potential distinction between the speed and accuracy components in learning motor or cognitive tasks.

Thus theoretical frameworks predominantly predict the positive effects of moderate-intensity exercise on cognition. Moreover, meta-analysis has pointed to a divergence of results when considering the effects of exercise upon speed and accuracy in various tasks. Based on these considerations as well as a variety of findings in studies that have examined the effects of aerobic exercise on motor learning, we conducted a study to examine the effect of moderate intensity aerobic exercise (ergometer cycling) on speed and accuracy task components in motor learning. In order to separate the speed and accuracy components, we applied a novel keyboard task (participants were not allowed to look at their fingers/keyboard) as a learning paradigm in order to capture both aspects. In the context of this task, speed is the number of letters produced in a specific time window and accuracy refers to the ability to produce correct words (i.e., hitting the right keys) during the same epoch.

## 2. Materials and Methods

### 2.1. Participants

Following approval of the experimental protocol by the regional ethics committee for medical research, 26 healthy participants were recruited from a university college community. Provided an average initial typing speed of 40 ± 15 words/min [30], it was estimated that a between-group standardized mean difference at 5% with 0.80 statistical power and an effect size of 0.30 would require 13 participants in each group. All participants reported to be healthy, without any neurological complaints, and were not receiving any form of medication/treatment. The study protocol was approved by the regional committee for medical and health research ethics (REC Central). All subjects provided informed written consent prior to participating in the study, and all procedures were carried out in accordance with the tenets of the Declaration of Helsinki.

### 2.2. Procedures

The pool of participants was divided into two groups by simple randomization (shuffled deck of cards): An exercise group (ergometer cycling) and a non-exercise control group in which the latter performed the same learning task without any previous exercise. The participants in the exercise group were all non-cyclists, however they reported familiarity with ergometer cycling as an exercise tool. The exercise and learning paradigm were conducted in a controlled lab environment three times a week for four weeks. In all sessions, the learning task was conducted immediately after exercising. All participants were assed at baseline, after 4 weeks (retest) and at 7-day retention.

### 2.3. Questionnaire

Information on demographics and physical activity level was obtained with the International physical activity questionnaire (IPAQ) including items on days, hours and the intensity of sport/exercise per week [31,32].

### 2.4. Cognitive Tasks

Cognitive abilities were assessed with the Letter-Number Sequencing (LNS) subtask from the Wechsler Adult Intelligence Scale (WAIS) [33] and with a visual spanboard task. The latter task consisted of a 5 × 5 grid presented on a screen and required participants to repeat patterns of flashing red circles with increasing complexity [34]. It has close similarity to n-back tasks typically applied for the assessment of working memory [35]. The test ended when two mistakes were made. The two tasks together are considered to capture the visuospatial sketchpad, central executive and phonological loop which are central components in the working memory construct [36,37].

### 2.5. Keyboard Typing

**Figure 1:**
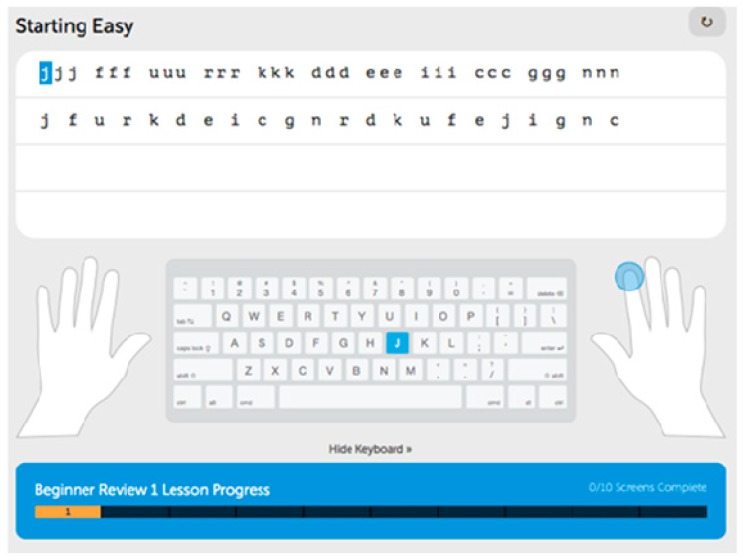
Screenshot with an example of a keyboard practice.

To investigate the effect of exercise on motor learning and retention, participants practiced keyboard typing on a portable laptop (13-inch screen, 28 cm × 11 cm keyboard) immediately after exercise or rest. The letters ‘F’ and ‘J’ contained kinesthetic markers. The task required typing without looking at the fingers/keyboard, following instructions provided on an open-source website [38] (see Figure 1). Next to the laptop, a picture of the keyboard set-up was provided. Each practice session consisted of 10 min of basic practice exercises on hitting the right keys provided by the software. As the learner completes various practices, they are moved to higher levels: The beginners level consists of practicing hitting specific keys three at a time (e.g., U, R, K), the intermediate level consist of common and easy words and sentences, and the advanced level consists of hitting the right keys for producing more complex sentences and paragraphs. Each practice trial was followed by a 3 min assessment of words typed per minute as well as the typing accuracy (%) in repeating a text. Throughout the course of the study, participants completed 120 min of keyboard practice.

### 2.6. Exercise

After a 5 min warm-up at a self-selected pace, participants in the exercise group completed a single 15 min bout of ergometer cycling. The intensity corresponded to ~65% of age-predicted maximal heart rate according to the formula 211 − 0.64 × age which has demonstrated excellent fit for participants in their twenties [39]. Exercise intensity was monitored by Polar M400s (Polar, Kempele, Finland). The duration and intensity of exercise applied was based upon meta-analytical data demonstrating that relatively short bouts of exercise (<20 min) with light-to-moderate intensity has the largest effect on memory [13].

### 2.7. Statistical Analysis

Kolmogorov–Smirnov tests, histograms and Q-Q plots were applied to confirm normality assumptions of the variable’s statistical distributions. Within and between-group effects were assessed with independent samples t-test, chi-square test, and Huynh-Feldt corrected repeated measures ANOVA. In all post hoc pairwise multiple comparisons, the alpha was Bonferroni corrected and the partial eta squared (*η2p*) was applied as a measure of effect size. Predictive Analytics Software (PASW, IBM, NY, US; previously SPSS) Version 25.0.0.1 was used for all statistical procedures with *p* < 0.05 as statistical significance criterion.

## 3. Results

Descriptive information regarding the study groups is provided in Table 1, which indicates that none of the demographical variables were significantly different between groups.

### 3.1. Speed of Keyboard Typing

**Figure 2:**
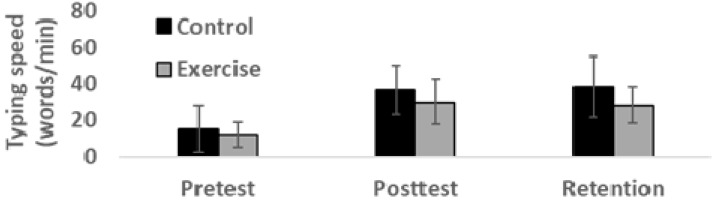
Mean (SD) keyboard typing speed for exercise and control group at pretest, posttest and 7-day retention tests.

As evident from Figure 2, there were no significant between-group differences in keyboard typing speed at pretest (*t* = 0.76, degrees of freedom (*df*) = 24, 95% CI for difference = −5.30–11.45, *p* = 0.46). The average baseline typing speed at 14 words per min can be seen as an indicator of the novelty of the task, as university students typically type (with vision) 2–3 times faster (Grabowski, 2008). Across the entire sample (*n* = 26) all participants improved their typing speed (words/min) from pre-test to post-test, an improvement that was maintained at the 7-week retention test. Consequently, the Huynh-Feldt corrected repeated measures ANOVA indicated a significant change in typing speed across the five-week study period (*F* = 92.79, *df* = 2, *p* = 0.001, *η2p* = 0.79). Post hoc analysis demonstrated a significant increase in typing speed from pre-test to post-test (95% CI for difference = 15.46–23.77, *p* < 0.001) and no significant difference from post-test to 7-day retention (95% CI for difference = −3.34–3.11, *p* > 0.05).

The exercise group had an average pre-test to post-test improvement of 109% and a 108% pre-test retention improvement in typing speed. Similarly, the experimental group improved (on average) their typing speed with 79% from pre-test to post-test and 77% from pre-test to retention. As evident from Figure 2, however, there were considerable individual differences in the magnitude of improvement, and the 2 (group) × 3 (time) Huynh-Feldt corrected repeated measures ANOVA indicated no significant group × time interaction effect (*F* = 2.23, *df* = 1, *p* = 15, *η2p* = 0.09).

### 3.2. Accuracy in Keyboard Typing

**Figure 3:**
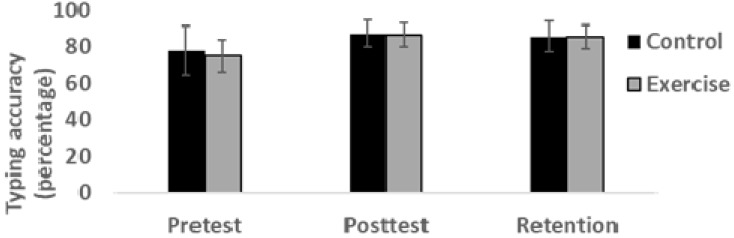
Mean (SD) keyboard typing accuracy for exercise and control group at pretest, posttest and 7-day retention tests.

The results for keyboard typing accuracy are depicted in Figure 3. Clearly visible, there were no significant between-group differences at pre-test (*t* = 0.64, *df* = 24, 95% CI for difference = 6.35–12.05, *p* = 0.53). Overall, the typing accuracy improved from pre-test to post-test, and improvement that was maintained at the 7-day retention test (*F* = 22.74, *df* = 2, *p* = 0.001, *η2p* = 0.49). Thus, post hoc analysis indicated significant improvement from pre-test to post-test (95% CI for difference = 4.99–15.49, *p* < 0.001) and no significant difference from posttest to 7-day retention (95% CI for difference = −3.23–0.91, *p* = 0.49).

The improvement in typing accuracy for the exercise group were on average 16% and 15% for pre-test to post-test and pre-test to retention, respectively. Similarly, control group participants improved by 14% from pre-test to post-test, and 12% from pre-test to 7-day retention. However, as for typing speed a substantial variability in individual improvement could be observed, and the 2 (group) × 3 (time) Huynh-Feldt corrected repeated measures ANOVA indicated no significant group × time interaction effect for improvement in typing accuracy (*F* = 0.18, *df* = 1, *p* = 68, *η2p* = 0.008).

## 4. Discussion

The principal aim of this current study was to examine the effect of moderate-intensity exercise on speed and accuracy task components in motor learning. To this end, participants (*n* = 26) were randomized to a non-exercise resting group (control) or an exercise group (ergometer cycling at 65% of age-predicted maximal heart rate). Immediately after exercise or resting, participants practiced a non-vision keyboard typing task through specialized online software. Both the exercise and control group improved substantially at both speed and accuracy in the non-vision keyboard typing task from pretest to posttest, a learning effect that was maintained at 7-day retention (see Figure 2 and Figure 3). The degree of improvement on both speed and accuracy task components, however, was not significantly different between the exercise and control group.

The results of the current study suggest that moderate-intensity aerobic exercise conducted successively before each practice (3 × week) across a four-week period do not improve the speed component more substantially in a keyboard typing task compared to simple resting before each practice (see Figure 2). This finding is in line with Singh et al. (2016) [20], in which applied exercise at moderate-intensity did not obtain any significant differences between the exercise group and control group on response time in a bimanual targeting task. Perini et al (2016) [22] however, found an improved speed of abduction movements with the left thumb (participants practiced moving as fast as possible) after ergometer exercise at 70% HR_max_. The perhaps strongest predictions from the cognitive-energetic approach [25,26], is that conducting moderate-intensity exercise leads to elevated arousal that optimize the availability of cognitive resources [29] and consequently, facilitate the learning of tasks that require substantial mental effort [27,28]. The increased arousal during moderate intensity exercise results in a faster information-processing speed, which is probably due to increased brain concentrations of the neurotransmitters dopamine and norepinephrine [4,5]. In our study, and those of Singh et al. (2016) [20] and Perini et al. (2016) [22], moderate-intensity exercises were indeed applied, which should have introduced an ‘optimal’ state of arousal and faster processing.

The similarity in exercise protocols in [22,24] and that of ours, thus points to differences in learning tasks as a potential source and explanation for differences in the study results. Indeed, our non-vision keyboard task and the task adopted by Singh et al. (2016) [22] shared the similarity that they required both speed and accuracy task components to be learned in order to improve on the tasks. This notion contrasts with the ‘speed-only’ task applied by Perini et al. (2016) [24]. It is possible to argue that the learning of more complex motor tasks is not (at least not to the same degree) facilitated by acute aerobic moderate-intensity exercise. Meta-analysis has pointed out that largest effect sizes are found for elementary cognitive tasks (e.g., reaction time) with relatively low task complexity and requiring mostly fast information processing [4,5]. Tasks with a higher complexity (such as our non-vision keyboard task) have been hypothesized to be susceptible to both positive and negative effects of acute exercise, as higher task complexity requires more prefrontal cortex activation than other tasks [40] and that the prefrontal cortex is more sensitive to exercise-induced arousal compared to other areas of the brain [41].

We did not find any increased learning rate or significantly better 7-day retention of task accuracy after four weeks of practicing the keyboard task, between the exercise group and control group (see Figure 3). In line with previous studies [20,21], it appears that moderate-intensity (60–70% of HF_max_) ergometer exercise with a 20 min–30 min duration do not facilitate the learning and retention of accuracy in motor tasks. This appear in contrast, however, to an overall positive effect on task accuracy in motor learning after high-intensity exercise [16,17,18]. This moderate vs. high intensity difference appears somewhat counterintuitive from a theoretical point of view, given that high exercise intensity might increase brain concentrations of the neurotransmitters norepinephrine and dopamine to a level that introduces neural noise, which could potentially have a negative effect on task accuracy (the catecholamines hypothesis: [26,42]). However, it is unknown what might constitute an ‘optimal’ level of neurotransmitter concentrations in the brain when engaging in learning tasks. Furthermore, high-intensity exercise requires greater activation of the premotor cortex and supplementary motor area. It has been stated that that these areas will be activated at the expense of the prefrontal cortex and induce poorer cognitive performance of complex tasks requiring prefrontal cortex activation [4]. One might speculate on an alternative explanation: Although these differences in brain activation might compromise cognitive processing, increased activation of motor areas might potentially facilitate motor learning and retention. Whether motor and cognitive tasks are differently affected by moderate or high-intensity exercise remains to be investigated in further studies.

Although the amount of available data on exercise-motor learning is somewhat moderate, the overall pattern of results seems to allow for the following working hypothesis: In motor tasks with predominant speed-requirements, an improvement in motor learning and retention can occur after moderate-intensity exercise (e.g., Perini et al., 2016 [22]). Motor learning and retention in more complex tasks, with conjunct speed and accuracy requirements on the other hand, is facilitated after high-intensity exercise [16,17,18] and not affected by moderate-intensity exercise as evident in the current study and that of others [21]. Exercise as a stressor might thus not have universal effects on motor learning/retention and may be dependent upon task and outcome measures [21,43]. Clearly, further studies should examine this hypothetical divergence between task complexity and the intensity of aerobic exercise. Establishing the theoretical underpinnings of such a hypothesis might also provide some refinement opportunities for the current models in the exercise-cognition literature.

The main limitation of the current study that warrants further examination is that we did not systematically include participants with different exercise habits and/or measure physiological capacity directly. Previously published data suggest that these might be important independent variables in the analysis of the interaction between exercise and cognition [1]. In this study, in which the participants were of similar age/BMI and reported similar levels of physical activity/sedentary behavior, the group that conducted exercise systematically before each motor learning session (3 × week for 4 weeks) did not outperform the non-exercise group on neither speed nor accuracy in a non-vision keyboard typing task. At the 1-week retention test, no differences were found between groups. These findings, and that of others, warrant further examination of the apparently complex relationship between various exercise components (intensity, task mode, duration, etc.) and the specific direction of learning effects upon various motor tasks and components (i.e., with different complexity).

## Figures and Tables

**Table 1:** Descriptive statistics across the two study groups. All values are mean (SD) unless otherwise reported.

	Study Group		
Variable	Exercise (*n* = 13)	Control (*n* = 13)	*p* ^1^
Male/Female (*n*)	4/9	7/6	0.70 ^2^
Age (years)	23.50 (2.54)	22.20 (2.78)	0.34
BMI (weight/height ^2^)	22.31 (2.45)	22.10 (2.52)	0.93
Spanboard	5.46 (1.13)	5.15 (0.80)	0.43
LNS	10.31 (3.04)	11.31 (1.84)	0.32
PA Level-leisure Moderate	75.5%	69.2%	0.50 ^2^
PA Level-leisure High	25.0%	30.8%	-
Sedentary time (hours)	8.91 (2.39)	9.23 (2.28)	0.74

^1^ Independent samples t-test, ^2^ Pearson Chi-Square. LNS: Letter Number Sequencing, BMI: Body Mass Index, PA: Physical Activity.
